# Neurocognitive Disorders and Their Factors in Chronic Consumers of Artisanal Alcohol, Tshitshiampa: A Study From Demba Territory, Central Kasaï Province

**DOI:** 10.7759/cureus.109346

**Published:** 2026-05-21

**Authors:** Pascal M Tshibuabua, Joseph N Tshitoko, Eric V Muteba, Guy M Bumoko

**Affiliations:** 1 Department of Neurology, University of Kinshasa, Kinshasa, COD; 2 Department of Psychiatry, University of Kinshasa, Kinshasa, COD

**Keywords:** artisanal alcohol, cognitive impairment, demba, drc, tshitshiampa

## Abstract

Introduction: Chronic consumption of the artisanal alcohol, tshitshiampa, is widespread in the territory of Demba, Democratic Republic of the Congo. This study aimed to evaluate the cognitive performance of chronic consumers of this beverage and to analyze the factors associated with cognitive impairment in this population.

Methods: A cross-sectional study was conducted among 202 chronic consumers of tshitshiampa. Alcohol consumption was assessed using the Fast Alcohol Consumption Evaluation score, and cognitive function was assessed using the Montreal Cognitive Assessment. Data were analyzed using Statistical Package for the Social Sciences version 27 (IBM Corp., Armonk, NY). Logistic regression analysis was performed to examine associations between cognitive performance and various explanatory variables. Statistical significance was set at p < 0.05.

Results: More than half of the participants (51.4%) presented cognitive impairment. The most affected cognitive domains were memory, orientation, executive functions, and visuospatial abilities. Cognitive impairment was significantly associated with higher levels of alcohol consumption, lower educational level, and the presence of alcohol-related comorbidities.

Conclusion: Chronic consumption of tshitshiampa is associated with a high prevalence of neurocognitive impairment. These findings highlight the importance of early cognitive screening and the implementation of prevention strategies adapted to the local socioeconomic context.

## Introduction

Excessive alcohol consumption constitutes a major public health problem worldwide [1]. While its somatic complications are well documented, its neurocognitive consequences remain insufficiently explored in many African contexts.

These consequences result from structural and functional alterations of neural circuits involved in executive functioning, memory processes, and social cognition [2]. For a long time, alcohol-related neurocognitive alterations were underestimated, as clinical attention was mainly focused on severe and well-characterized conditions such as Korsakoff syndrome, Marchiafava-Bignami disease, or alcoholic dementia [3].

With the evolution of diagnostic classifications, the introduction of substance-induced major and mild neurocognitive disorders has made it possible to recognize the existence of earlier, more frequent, and sometimes reversible cognitive deficits.

In several international studies, more than half of chronic alcohol consumers present some degree of neurocognitive impairment. Cognitive deficits are reported in approximately 50%-80% of alcohol-dependent individuals and typically affect multiple domains, particularly executive functions and episodic memory, followed by attention and visuospatial abilities, while language functions remain relatively preserved [4,5]. Clinical studies conducted in patients with alcohol use disorders further report that a substantial proportion of individuals exhibit impairment in at least one cognitive domain, with significant consequences for daily functioning and treatment outcomes [6].

However, most of the available data originate from hospital-based populations, which may limit their generalizability to community settings. In Africa, research examining the cognitive impact of alcoholic beverages remains scarce. The few available studies report a high prevalence of neurocognitive impairment among consumers of traditional alcoholic beverages [7,8].

In the Democratic Republic of Congo, particularly in the territory of Demba (Central Kasai Province), tshitshiampa is a widely consumed artisanal alcoholic beverage. Due to its low cost, this drink, produced by distilling fermented maize and cassava, is highly accessible to all segments of the population, especially in disadvantaged neighborhoods. The recent multiplication of sales points has contributed to increased consumption in a socioeconomic context characterized by unemployment, illiteracy, and poverty. These factors favor alcohol dependence and may contribute to a reduction in cognitive reserve, thereby increasing vulnerability to neurocognitive disorders [9,10].

Despite this widespread consumption, no local study has yet evaluated the cognitive profile of chronic consumers of tshitshiampa. In this context, the main objective of this study is to assess the cognitive performance of these consumers and to identify factors associated with cognitive impairment.

More specifically, it aims to describe the sociodemographic characteristics of regular consumers of tshitshiampa, to analyze their levels and habits of alcohol consumption, and to examine the relationship between chronic consumption of this substance and the onset of neurocognitive disorders.

The study also seeks to determine the cognitive domains most affected by chronic alcohol exposure, as well as to assess the influence of the degree of alcohol consumption on cognitive performance. Furthermore, it investigates the potential role of associated comorbidities and educational level in the development of these disorders. Such an approach not only fills a significant scientific gap but also provides valuable information for planning appropriate public health interventions.

## Materials and methods

Study design and setting

This descriptive cross-sectional study was conducted among regular consumers of the artisanal alcoholic beverage tshitshiampa residing in Demba. The planned study period was from June 6, 2025, to June 6, 2026, covering all aspects of the research, including formulating the research problem, field data collection, and processing and analysis of results. However, the actual data collection phase took place from April 1, 2026, to May 3, 2026.

Demba is a territory located in the central Kasai Province of the Democratic Republic of Congo. Administratively, it is divided into seven sectors (Lusonge, Diofwa, Bena Mamba, Mwanzangoma, Tshibote, Tshibungu, and Lombelu) and two rural communes (Demba and Bena-Leka).

The region is characterized by a high production of artisanal alcoholic beverages, notably tshitshiampa, which is widely consumed by the local population. It is the main, if not the only, alcoholic beverage available in the region, as industrial beer is both expensive and rarely accessible. The composition of this artisanal and unregulated beverage remains unknown. However, it is possible that it contains other potentially neurotoxic substances in addition to ethanol. The widespread consumption of tshitshiampa thus represents a major public health issue.

Study population

Inclusion Criteria

Participants were eligible for inclusion if they met all of the following criteria: age ≥ 18 years, residence in one of the selected villages of the Demba territory, chronic consumption of tshitshiampa for at least 12 consecutive months prior to enrollment, ability to communicate adequately and provide informed consent, absence of major visual or hearing impairment likely to interfere with neurocognitive assessment, and being sober and free from acute alcohol intoxication at the time of the interview and cognitive evaluation.

Exclusion Criteria

Participants were excluded if they met at least one of the following criteria: refusal to participate or inability to provide informed consent; acute alcohol intoxication at the time of assessment; severe visual, hearing, or communication impairments preventing proper administration of the study instruments; history of previously diagnosed major neurocognitive disorders, including Alzheimer’s disease, vascular dementia, frontotemporal dementia, or any other established form of dementia; history of previously diagnosed neurocognitive disorders or severe neurological diseases likely to independently affect cognitive performance; presence of severe psychiatric disorders or altered mental status interfering with reliable cognitive evaluation; chronic use or dependence on other psychoactive substances likely to influence cognitive functioning; or presence of any medical or neurological condition judged by the investigators to significantly compromise the validity of cognitive assessment.

Data collection

A two-stage cluster sampling method was employed. The Demba territory comprises seven sectors and two rural communes. Two sectors were randomly selected. Within these sectors, the following villages were randomly chosen: Bena Masona and Bakua Mpika (Tshibote sector); Bena-Mbala and Basangana (Bena Mamba sector).

A total of 23 tshitshiampa sales and consumption sites were identified, resulting in the identification of 242 consumers. Of these, 231 agreed to participate. After applying the inclusion criteria, a final sample of 202 participants was retained for analysis.

Recruitment and data collection procedure

Data collection was conducted by a team of four physicians, including three general practitioners and one neurology resident. Data were collected using a structured questionnaire specifically developed for this study to capture sociodemographic characteristics, alcohol consumption patterns, medical history, and comorbid conditions. Alcohol consumption was assessed using the Fast Alcohol Consumption Evaluation (FACE) questionnaire in French, a validated screening tool for alcohol use disorders and a freely available instrument. This questionnaire was selected due to its simplicity, feasibility in low-resource settings, and prior use in similar populations [11]. The general practitioners had received prior training in administering the FACE questionnaire. The neurology resident is trained and certified by the Montreal Cognitive Assessment (MoCA) team for the standardized administration and scoring of the MoCA.

Cognitive performance was evaluated using the MoCA version 8.3 in French, administered in accordance with the official administration guidelines. One point was added to the total MoCA score for participants with fewer than 12 years of education [12]. Prior to its implementation, formal authorization for use of the MoCA in this study was obtained from the MoCA team. This test was administered face-to-face in a quiet environment. Each item was scored according to the official MoCA administration. The total score was used to quantify cognitive performance.

Study variables

Independent Variables

The independent variables include sociodemographic characteristics (age, sex, marital status, years of education, and employment status); alcohol consumption characteristics (duration, frequency, quantity, and FACE score); comorbidities (hepatic, neurological, and psychiatric disorders, as well as family history); and prenatal alcohol exposure.

Dependent Variables

The cognitive performance was assessed using the MoCA score and the total MoCA score.

Statistical analysis

Data were analyzed using Statistical Package for the Social Sciences software (version 27.0; IBM Corp., Armonk, NY). Descriptive statistics were used to summarize the general characteristics of the study population, including sociodemographic, clinical, and behavioral variables. The distribution of FACE and MoCA scores was described using frequencies, percentages, and tabular presentations.

Multivariate logistic regression analysis was performed to identify factors associated with impaired cognitive performance. Variables considered clinically relevant or associated with cognitive impairment in previous literature were included in the multivariate logistic regression model to control for potential confounding effects. These variables included age, sex, educational level, occupational status, alcohol dependence severity (FACE score), family history of alcoholism, and presence of alcohol-related comorbidities when applicable. Adjusted odds ratios with 95% confidence intervals were calculated to identify independent factors associated with cognitive impairment. Variables with a p value of <0.20 in univariate analysis were considered eligible for inclusion in the multivariate model. Statistical significance was set at p < 0.05.

Ethical considerations

The objectives of the study were clearly explained to all participants prior to enrollment, and written informed consent was obtained. Participation was entirely voluntary, and participants were free to withdraw at any time without any consequences.

Core ethical principles, including confidentiality, autonomy, and beneficence, were strictly upheld throughout the study. The study posed no direct risks or benefits to participants.

The research protocol was approved by the Ethics Committees of the Ministry of Public Health (Approval No. 670/CNES/BN/PMMF/2025, dated June 6, 2025) prior to the initiation of data collection.

Participants identified as having severe cognitive impairment were referred to appropriate healthcare services for further evaluation and management.

## Results

This section presents the findings obtained from 202 chronic consumers of tshitshiampa residing in the territory of Demba.

Sociodemographic characteristics of the participants

The study population was predominantly men (75.7%), with a sex ratio of 3.1 in favor of men. The mean age of participants was 31.7 ± 11 years, with a predominance of young adults aged 18-27 years (39.1%). Regarding marital status, married individuals (48.5%) and single participants (44.5%) were similarly represented. Participants with fewer than 12 years of education constituted the majority of the sample. From an occupational perspective, 40.1% of participants were unemployed (Table 1).

**Table 1:** Distribution of participants according to sociodemographic characteristics (n = 202)

Parameter	Frequency	Percentage
Age		
18-27	79	39.1
28-37	62	30.6
38-47	41	20.2
48-57	20	9.9
Sex		
Male	153	75.7
Female	49	24.3
Marital status		
Single	90	44.5
Divorced	7	3.4
Married	98	48.5
Widowed	7	3.4
Years of education		
Under 12 years	128	63.3
More than 12 years	47	36.6
Occupational status		
Traveller/transporter	32	15.8
Miner	24	11.8
Farmer	41	20.2
Small trader	24	11.8
Unemployed	81	40.1
Total	202	100

Characteristics of tshitshiampa consumption

All 202 participants (100%) reported consuming tshitshiampa for more than one year. The distribution of participants according to the FACE questionnaire score indicated a high prevalence of alcohol misuse and dependence within the study population (Table 2).

**Table 2:** Distribution according to FACE questionnaire score (n = 202) FACE: Fast Alcohol Consumption Evaluation

Category	Frequency	Percentage
Alcohol dependence (FACE ≥9)	42	20.8
Alcohol misuse		
Women		
Score <4	31	15.3
Score ≥4	16	7.8
Men		
Score <5	46	22.9
Score ≥5	67	33.2
Total	202	100

Cognitive performance of consumers

More than half of the chronic tshitshiampa consumers (51.4%) presented cognitive impairment according to the MoCA score (MoCA < 26) (Table 3).

**Table 3:** Distribution according to MoCA score (n = 202) MoCA: Montreal Cognitive Assessment

MoCA score	Frequency	Percentage
≥26/30	98	48.5
18-25	50	24.7
10-17	42	21.0
<10	12	5.7
Total	202	100

All cognitive domains assessed were affected, with memory being the most impaired domain, as 160 participants (79.2%) scored below the normal threshold. In contrast, language functions appeared relatively better preserved compared with other cognitive domains (Table 4).

**Table 4:** Distribution according to impaired cognitive domains

Cognitive domain	Score	Frequency	Percentage
Visuospatial/executive	<5	115	56.9
	5	87	43.0
Naming	<3	107	53.0
	3	95	47.0
Attention	<6	114	56.4
	6	88	43.5
Language	<3	70	34.8
	3	132	65.3
Abstraction	<2	130	64.4
	2	72	35.6
Memory	<5	160	79.2
	5	42	20.7
Orientation	<6	154	76.2
	6	48	23.6

Factors associated with cognitive impairment

Multinomial logistic regression analysis revealed significant associations between impaired neurocognitive performance and several variables, including: alcohol dependence level (FACE score); male sex; lower educational level; family history of alcoholism; and alcohol-related comorbidities. These associations remained statistically significant after multivariate adjustment (Table 5).

**Table 5:** Factors associated with cognitive impairment (MoCA < 26): multivariate logistic regression (n = 202) MoCA: Montreal Cognitive Assessment; OR: odds ratio; CI: confidence interval; FACE: Fast Alcohol Consumption Evaluation

Variables	Crude OR	95% CI	p	Adjusted OR	95% CI	p
Male sex	3.88	1.81-6.47	<0.001	2.22	1.11-4.16	0.024
Age (per year)	1.04	1.00-1.04	0.041	1.01	0.96-1.03	0.118
Low education level	3.04	1.49-4.89	<0.001	2.21	1.10-4.22	0.025
Family history of alcoholism	3.95	2.13-6.99	<0.001	3.09	1.58-5.98	0.001
Presence of comorbidity	3.34	1.52-5.79	<0.001	2.34	1.12-4.84	0.022
Severe dependence (FACE ≥ 9)	5.71	2.41-12.10	<0.001	3.74	2.21-10.10	<0.001

## Discussion

The analysis of cognitive performance among the 202 chronic consumers of tshitshiampa in Demba reveals a high prevalence of cognitive impairment. More than half of the participants (51.4%) presented an MoCA score compatible with cognitive impairment. This proportion is comparable to those reported in several studies examining chronic alcohol consumption. Studies conducted in Europe and North America report prevalences ranging from 50% to 80% of cognitive deficits among individuals with alcohol dependence [4,5].

The impairments observed primarily affect memory, executive functions, attention, and visuospatial abilities, domains known to be particularly sensitive to alcohol-related neurotoxicity. The predominance of memory impairment among individuals with alcohol use disorders has been widely described in the literature. Impairments in episodic memory represent one of the central markers of cognitive disorders in alcohol-dependent individuals and may persist despite abstinence, forming the basis of severe conditions such as Korsakoff syndrome [2,13].

Language functions appeared to be relatively better preserved. This observation is consistent with the literature, indicating that basic language abilities, including comprehension and simple naming, remain preserved for a longer period in alcohol-related cognitive disorders, unlike memory and executive functions, which are affected earlier [2]. The cognitive profile observed in our study is therefore consistent with that described in alcohol-related neurocognitive disorders.

Cognitive impairment and sociodemographic characteristics

Age

Cognitive decline among chronic consumers of tshitshiampa appears to occur regardless of age. The majority of the sample consisted of young adults, who already exhibited high proportions of altered MoCA scores, suggesting an early onset of cognitive decline associated with chronic consumption of artisanal alcohol.

Studies conducted among young heavy alcohol consumers have also demonstrated early cognitive impairments, suggesting a particular vulnerability of the young brain to the neurotoxic effects of alcohol [14]. This observation highlights the importance of early screening and preventive strategies, particularly among young individuals, in order to prevent progression toward more severe forms of neurocognitive disorders.

Sex

Men showed a higher proportion of cognitive impairment than women. This observation is consistent with the structure of the sample, which was largely dominated by men, representing 75.7% of chronic tshitshiampa consumers. The higher proportion of cognitive impairment observed among men in this study reflects a dual mechanism: greater alcohol exposure and more vulnerable socioeconomic conditions.

These results are generally consistent with the international literature, which identifies men as a group at particularly high risk for developing alcohol-related neurocognitive disorders, especially in young and socioeconomically disadvantaged populations. Studies conducted in sub-Saharan Africa indicate that men consume alcohol more regularly and in larger quantities than women, thereby increasing their exposure to neurocognitive complications [15]. Mann et al. also demonstrated sex-related differences in brain vulnerability to alcohol, suggesting that men may have an increased risk of cognitive decline due to biological, behavioral, and sociocultural factors [16].

Level of Education

Educational level appears to be an important determinant. Individuals with lower educational attainment showed higher proportions of cognitive impairment, whereas participants with university-level education were proportionally less affected.

This observation aligns with the theory of cognitive reserve, which suggests that individuals with higher educational attainment may benefit from greater resistance to the neurocognitive effects of alcohol. A strong relationship exists between low sociocultural level (assessed by years of education) and the prevalence and severity of cognitive impairment [2]. The authors relate these findings to the concept of cognitive reserve, which allows individuals to maintain normal cognitive functioning despite the occurrence of deleterious events.

Occupation

Workers engaged in physically demanding, poorly paid occupations, such as mining and farming, showed higher rates of neurocognitive impairment, whereas civil servants and small-scale traders demonstrated better cognitive performance.

These differences likely reflect the combined effects of chronic alcohol consumption, difficult working conditions, chronic socioeconomic stress, and limited access to healthcare resources, prevention programs, and harm reduction services.

Cognitive impairment and characteristics of alcohol consumption

All participants had been consuming tshitshiampa for more than one year, with 57.7% reporting consumption at least five times per week. Both the frequency and quantity of alcohol consumption significantly increased the probability of a pathological MoCA score.

These findings are consistent with previous studies demonstrating a dose-response relationship between alcohol consumption and cognitive decline [17]. Chronic exposure to alcohol has been shown to induce widespread deficits in memory, executive functions, and attention [4].

Cognitive impairment and family history of alcoholism

The results of this study demonstrate a significant association between a family history of alcohol consumption and the prevalence of cognitive impairment among tshitshiampa consumers. This relationship suggests a cumulative and intergenerational effect of alcohol-related behaviors, where familial exposure represents both a biological, cultural, and environmental risk factor. Familial aggregation of alcoholism is an important predictive factor, pointing toward an interaction between genetic predisposition and the family environment.

Previous studies have shown that alcohol use disorders are moderately heritable, with genetic factors accounting for nearly 50% of risk [18]. Furthermore, individuals with a family history of alcoholism may exhibit early neurocognitive alterations even before the onset of dependence [19].

Family history of alcohol consumption, therefore, constitutes a major risk factor for the development of neurocognitive impairment in the population of Demba. It influences not only the initiation of alcohol use but also the severity of cognitive decline by amplifying the neurotoxic effects of tshitshiampa. This finding highlights the need for targeted interventions among at-risk families, particularly through early prevention programs, community education initiatives, and monitoring of exposed youth.

Cognitive impairment and comorbidities

Statistical analysis revealed a significant association between the presence of comorbidities and cognitive impairment, indicating that individuals presenting at least one alcohol-related condition (such as liver disease, peripheral neuropathy, gastrointestinal disorders, or malnutrition) showed proportionally higher rates of altered MoCA scores.

These comorbidities may amplify the neurotoxic impact of alcohol through both direct and indirect mechanisms. This phenomenon is particularly relevant in the context of tshitshiampa, an artisanal beverage that may contain elevated levels of additional toxic substances such as methanol and mycotoxins, both of which have well-established neurotoxic effects.

The higher proportion of cognitive impairment observed among individuals with comorbidities also reflects the cumulative effect of chronic medical conditions that weaken brain structures and reduce cognitive reserve.

These findings are consistent with international literature demonstrating that the presence of comorbidities, particularly metabolic diseases, nutritional disorders, and neurological conditions, significantly increases the likelihood of cognitive deficits among individuals with alcohol dependence [20].

Regional context and socioeconomic impact

The results observed in Demba are consistent with a broader trend reported in Central Africa, where the consumption of similar artisanal alcoholic beverages has been associated with significant cognitive impairment, particularly in cities such as Lubumbashi [21]. Similar findings have also been reported with other locally produced neurotoxic substances such as dolo and marula beer [7,8]. Artisanal alcoholic beverages may contain toxic contaminants such as methanol, which are associated with severe neurological damage [22].

The social and personal impact of these disorders is considerable, including reduced productivity due to mental health problems, family disruption associated with caregiving for individuals with cognitive disorders, increased pressure on healthcare systems, and the exacerbation of poverty cycles. These findings underscore the urgent need for targeted public health interventions [23].

## Conclusions

Chronic consumption of the artisanal alcoholic beverage tshitshiampa is associated with a high prevalence of neurocognitive impairment, affecting more than half of consumers in the studied population. The most impacted domains include memory, executive functions, orientation, and visuospatial abilities, highlighting the diffuse nature of alcohol-related brain dysfunction. Several factors, including the level of alcohol dependence, low educational attainment, male sex, and the presence of comorbidities, were significantly associated with impaired cognitive performance.

These findings underscore the urgent need for systematic cognitive screening among chronic alcohol consumers, particularly in low-resource settings where artisanal alcohol use is widespread. Preventive strategies, early detection, and targeted interventions should be implemented to reduce the burden of neurocognitive disorders and improve long-term outcomes in these vulnerable populations.
